# Zinc sulfate acts as an efflux pump inhibitor on *Pseudomonas aeruginosa* clinical isolates

**DOI:** 10.1007/s11274-025-04352-4

**Published:** 2025-04-28

**Authors:** Wedad M. Abdelraheem, Hadeer Ibrahim Yassin, Shaimaa Zaki, Mona Abdel Monem Esmail

**Affiliations:** Department of Medical Microbiology and Immunology, Faculty of Medicine, Minia University, Minia, Egypt

**Keywords:** *Pseudomonas aeruginosa*, Efflux pump, Antimicrobial resistance, Gene expression

## Abstract

Efflux pumps in *Pseudomonas aeruginosa* play an important role in decreasing the bacterium’s antibiotic susceptibility. This study aimed to identify *P. aeruginosa* efflux pumps and examine the effect of Zinc sulfate on these pumps. This study was conducted on 104 *P. aeruginosa* clinical isolates collected from different types of specimens. The antimicrobial susceptibility pattern of all isolates was examined by disc diffusion and microdilution methods. A Cartwheel test was done for phenotypic detection of *P. aeruginosa* efflux pumps in the presence and absence of zinc sulfate. The expression of efflux pumps encoding genes and their regulators in the presence and absence of zinc sulfate was measured using real-time reverse transcriptase PCR. A high rate of multidrug-resistant (MDR) pattern was observed among the tested isolates (55.7%). Combining different antibiotics with Zinc sulfate exhibited synergistic effects against most of the studied isolates. The addition of sub-inhibitory concentrations of Zinc sulfate to the cartwheel test was found to inhibit efflux pump activity in all tested isolates. The expression of the positive regulator gene, *mexT*, was significantly decreased. Additionally, Zinc sulfate significantly upregulated the expression of the *mexR* gene (P-value < 0.05), a negative regulator of the MexAB-OprM efflux pump. Zinc sulfate demonstrated a strong antimicrobial effect against *P. aeruginosa* clinical isolates. We recommend the use of Zinc sulfate as an antibiotic adjuvant and efflux pump inhibitor in the treatment of *P. aeruginosa* infections to enhance the bacterium’s susceptibility to antimicrobial agents.

**Supplementary Information:**

The online version contains supplementary material available at 10.1007/s11274-025-04352-4.

## Introduction

*Pseudomonas aeruginosa* is an opportunistic pathogen of extreme clinical importance worldwide and a common cause of nosocomial infections with a high rate of morbidity and mortality (Choi et al. 2018)***.**** P. aeruginosa* can resist many of the currently available antibiotics through three types of resistance: intrinsic, acquired, and adaptive It has intrinsic resistance to various antibiotics due to factors such as reduced outer membrane permeability, the production of antibiotic-inactivating enzymes, and the presence of efflux pumps (Pang et al. 2019).

Acquired resistance can occur through the overexpression of efflux pumps and the production of β-lactamases or as a result of horizontal gene transfer of antibiotic-resistant genes (Meletis and Bagkeri 2013). Adaptive antibiotic resistance of *P. aeruginosa* includes biofilm-mediated resistance and the formation of antibiotic-tolerant persister cells (Colclough et al. 2020; Coleman et al. 2020).

Efflux pumps are transport proteins that help remove toxic substances from bacterial cells, which leads to antibiotic resistance. These pumps are present in all bacterial species, particularly gram-negative bacteria (Zwama and Nishino 2021). In *P. aeruginosa*, six efflux pumps are involved in antimicrobial resistance: MexAB-OprM, MexJK-OprM, MexEF-OprN, MexXY-OprM, MexCD-OprJ, and MexVW-OprM (Alcalde-Rico et al. 2018).

Antimicrobial resistance can be reduced by developing efflux pump inhibitors (EPIs), which are an approach to combat antibiotic resistance. By preventing the action of efflux pumps, EPIs can restore the effectiveness of antibiotics, allowing them to accumulate within bacterial cells to reach the minimal bactericidal concentration and effectively combat the infection. (Rao et al. 2018).

Zinc sulfate is used in the management of diarrheal diseases as oral supplement. Zinc salts have shown inhibitory effects against a wide range of bacterial species, such as *Salmonella*, enteropathogenic *Escherichia coli*, Shigella and *Vibrio cholerae,* as well as *Bacillus cereus,* Lactococcus*, Klebsiella pneumoniae, Proteus mirabilis and S. aureus* through destructions of bacterial cell wall and ROS production (Gonçalves et al*.* 2017). The antibacterial effects were observed to follow the order of Zn^2^⁺ > Cu^2^⁺ > Ag⁺ > Al^3^⁺ in a 100 mL sulfate solution during the halo inhibition tests, with Zn^2^⁺ ions demonstrating the most significant effect among the sulfates (Ishida 2023). So, in this study, we aimed to determine the percentage of positive efflux pumps among MDR *P. aeruginosa* clinical isolates. Also, we aimed to evaluate Zinc sulfate as an efflux pump inhibitor.

## Methods

### Study design

This study was performed from February 2022 to June 2023 in the Department of Medical Microbiology and Immunology, Faculty of Medicine, Minia University, Egypt. Three hundred and fifty specimens were gathered from patients of the gynecological, General, and Plastic surgery departments, and ICUs at Minia University Hospitals. All age groups above 5 years are included in the study. Specimens were obtained from patients complaining of UTI with painful micturition and fever, Wound infection with redness, edema, and pus formation, or burn wound infections. Males and Females were included.

### Bacterial isolation and identification

The specimens were plated on cetrimide agar (Oxoid, England) and incubated at 37 °C overnight. After incubation, the isolated colonies were identified through microscopic examination and biochemical reactions, that are the oxidase test, citrate test, and triple sugar iron agar test (Cheesbrough 2000). The strains confirmed as *P. aeruginosa* were cultured in nutrient broth (Oxoid, UK), incubated for 24 h, then stored in 20% glycerol and frozen at − 20 °C for subsequent analysis.

### Chemicals and media

- Antimicrobial discs: Cefepime (30 μg), Ceftazidime (30 μg), Piperacillin (100 μg), Gentamycin (10 μg), Amikacin (10 μg) and Levofloxacin (5 μg). All discs were obtained from Oxoid Company, UK.
- Muller-Hinton (MH) agar, Oxoid Company, UK.
- Muller Hinton Broth, Oxoid Company, UK.
- Antibiotics powders: Meropenem (MYLAN S.A.S, France), Colistin sulfate (Epico Egypt), and Piperacillin-Tazobactam (Pfizer USA).
- Zinc sulfate (Epico–Pharma, Egypt).
- Ethidium Bromide (Thermo Fisher Scientific, USA).
- Trypticase Soy Agar (TSA), Oxoid Company, UK.

### Antimicrobial susceptibility testing

Antibiotic susceptibility testing was performed on all isolates by the disc diffusion method against the following antimicrobial discs: Cefepime (30 μg), Ceftazidime (30 μg), Piperacillin (100 μg), Gentamycin (10 μg), Amikacin (10 μg) and Levofloxacin (5 μg). *P. aeruginosa* ATCC 27853 was included as a positive control. The results were interpreted following CLSI 2022.

Antimicrobial susceptibility testing by microdilution method was performed using the following antibiotic powders: Meropenem, Colistin sulfate, and Piperacillin-Tazobactam according to CLSI 2022 guidelines (CLSI 2022).

### Detection of zinc sulfate MIC By broth microdilution

The stock solutions of zinc sulfate were performed at concentrations of at least (8192 μg/ml).by using distilled water. The stock was used on the same day. Using the direct colony suspension technique, bacterial pure colonies inoculum was prepared. Antimicrobial effect of zinc sulfate was evaluated by broth microdilution method in 96 well ELISA plate, then the plate was incubated for 18–24 h. The lowest zinc concentration that inhibits visible microorganism growth was determined.

### MIC detection of zinc sulfate combined with antibiotics

Zinc sulfate was combined with each of 6 tested antibiotics (Ceftazidime, Gentamycin, Levofloxacin, Meropenem, Colistin sulfate, and Piperacillin/Tazobactam) against the study isolates. MIC was evaluated by broth microdilution method. The Fractional inhibitory concentration index (FICI) was calculated to interpret the results of zinc sulfate combination with different antibiotics tested as follows (Bellio et al. 2021): FICI = FICA + FICB

$$\text{FICA (for antibiotic)}=\frac{\text{MIC of agent A in combination }}{\text{MIC A alone}}$$

$$\text{FICB }\left(\text{for Zinc sulfate}\right)=\frac{\text{MIC of agent B in combination }}{\text{MIC B alone}}$$

If the result of the FICI is less than or equal to 0.5, it is considered synergy; if it is > 4.0 it is considered antagonism; if it is between > 0.5 and ≤ 1.0 it is an additive result, and if it is between > 1 and ≤ 4.0, it is considered as indifference (Bellio et al. 2021).

### Phenotypic detection of efflux pumps by ethidium bromide cartwheel assay

Ethidium Bromide (EtBr) in the cartwheel assay is used to detect the activity of bacterial efflux pumps depending on the passage of EtBr over the cytoplasmic membrane and subsequent intracellular accumulation within the bacterial cell. In brief, Trypticase Soy Agar (TSA) plates supplemented with different EtBr concentrations ranging from (0, 1, 1.5, 2, and 2.5 mg/l) were prepared and the tested isolates were streaked in a cartwheel pattern on these plates. *P. aeruginosa* PAO1 was used as a reference positive control strain. The plates were incubated aerobically at 37 °C for 24 h. After 24 h incubation, the plates were observed under UV light in a UV transilluminator for efflux activity**.** The streaked isolates with no fluorescence at ≥ 0.5 mg/l EtBr concentration were considered efflux pump positive and those with clearly visible fluorescence at 0.5 mg/l EtBr concentration were considered efflux pump negative. The efflux pump activity was further classified as mild (no fluorescence at 0.5–1 mg/l, EtBr conc.), moderate (no fluorescence at 1.5 mg/l, EtBr conc.), or High (no fluorescence at ≥ 2 mg/l, EtBr conc) (Martins et al. 2013).

### Phenotypic detection of zinc sulfate effect in *P. aeruginosa* efflux pump

Ethidium Bromide (EtBr) in the cartwheel assay is used again to detect the zinc sulfate effect on the efflux pump by adding 1/4 MIC of zinc sulfate (128 µg/ml) to all MDR *P. aeruginosa* strains broth and incubated at 37 °C for 6 h. Then the broth of the tested strains was streaked on Trypticase Soy Agar (TSA) plates supplemented with different EtBr concentrations and the procedure was completed as mentioned above.

### Genotypic detection of efflux pumps encoding genes and their regulators by conventional PCR

The presence of genes encoding four different efflux pumps most involved in *P. aeruginosa* antibiotic resistance and their regulator genes were examined in all MDR isolates using the conventional PCR method as listed in Table 1(Murugan et al. 2018; Hassuna et al. 2020). At first, the DNA of *P. aeruginosa* isolates was extracted by the heat shock method as previously described (Dashti et al. 2009)**.** The PCR amplification was carried out in 50 µl reactions as follows: in Eppendorf 25 µl COSMO PCR Hot Start RED Master Mix, 5 µl of extracted DNA, and 1 M of each primer. The reaction was completed by PCR water. The PCR amplification was carried out in Biometra UNO II thermal cycler (Goettingen, Germany) under the following conditions: initial denaturation at 94 °C for 5 min, then 35 amplification cycles (denaturation 30 s at 94 °C, annealing temperature varies according to different genes, and extension 30 s at 72 °C), and a final extension at 72 °C for 5 min. Amplified products were analyzed by electrophoresis in 2% agarose gel at 80 V for 45 min in Tris–borate–EDTA buffer containing ethidium bromide under ultraviolet irradiation.

**Table 1:** Primer sequences, annealing temperatures, and amplicon sizes of tested genes

Efflux pumps	Gene tested	Primer sequence	Annealing temp. (°c)	Amplicon size (bp)
MexAB-OprM	*mexA*	F: AACCCGAACAACGAGCTG	52	950
		R: ATGGCCTTCTGCTTGACG		
	*mexB*	F: GTGTTCGGCTCGCAGTACTC	57	244
		R: AACCGTCGGGATTGACCTTG		
	*oprM*	F: TACCAGAAGAGTTTCGACCTGAC	59	812
		R: CATGTGTCAAAACAGTCACCTCC		
Regulator (-ve)	*mexR*	F: CCGTGAATCCCGACCTGATG	55	450
		R: TGACATGATGGCTTCCGCAT		
carbapenem porin	*oprD*	F: CTACGGCTACGGCGAGGAT	57	35
		R: GACCGGACTGGACCACGTACT		
MexCD-OprJ	*mexC*	F: GGAAGAGCGACAGGAGGC	57	175
		R: CTGCACCGTCAGGCCCTC		
Regulator (-ve)	*nfxB*	F: TGACCCTGATTTCCCATGACG	55	344
		R:GTAGACCAGGGTGATGAACAGT		
MexEF-OprN	*mex E*	F: TACTGGTCCTGAGCGbCCT	56	130
		R: TCAGCGGTTGTTCGATGA		
Regulator (+ve)	*mexT*	F: TGC ATC ACG GGG TGA ATA AC	55	1398
		R: GGT AGC GCC AGG AGA AGT G		
MexXY- OprM	*mex X*	F: GGCTTGGTGGAAGACGTG	53	750
		R: GGCTGATGATCCAGTCGC		
	*mex Y*	F: CCGCTACAACGGCTATCCCT	57.5	246
		R: AGCGGGATCGACCAGCTTTC		
Regulator (-ve)	*mexZ*	F:ATTGGATGTGCATGGGTG	55	1000
		R: TGGAGATCGAAGGCAGC		
Reference gene	*Rspl*	F: GCAACTATCAACCGACTGGTG	57	241
		R: GCTGTGCTCTTGCAGGTTGTG		

### Expression of efflux pumps encoding genes and their regulators in *P. aeruginosa* isolates (in the presence and absence of zinc sulfate application

The expression of efflux pumps encoding genes and their regulators in all *P. aeruginosa* isolates reported as positive for the corresponding gene by conventional PCR were examined by relative gene expression method before and after zinc sulfate application according to the following steps:

#### RNA extraction

Pure bacterial colonies were inoculated in two tubes containing 2 ml nutrient broth. One of the tubes contained sub-MIC of zinc sulfate. Tubes were incubated at 37 °C for 24 h**.** Bacterial RNA was extracted using the Direct-zol RNA extraction kit (Zymo Research CORP, Australia) according to the manufacturer’s instructions. The presence of the extracted RNA was examined via electrophoresis on 1.2% agarose gel at 100 V for 60 min.

#### Reverse transcriptase PCR (RT-PCR)

Quantitative real-time rt-PCR was performed using one-step SYBR green kits (HERA PLUS SYBR® Green qPCR Kit–Willowfort) according to manufacturer instructions in an ABI 7500 instrument (Applied Biosystems, USA)**.** Negative control specimens containing water instead of template, one for each gene were included in the same PCR run. RT-PCR results were analyzed with relative quantification to *the rpsL* gene used as a reference gene. The relative fold change was calculated using the delta CT method (Livak and Schmittgen 2001).

### Statistical analysis

All data collected in this study were stored in a computer database. Statistical analysis was done on IBM SPSS package version 22 (SPSS Inc., Chicago, IL, USA). Chi-squared tests were performed for categorical data, while paired specimen T-tests were used for the calculation of the P-value of means. Wilcoxon test was used for comparing the non-parametric variables. A significant level of P-value is < 0.05.

## Results

A total of 104 *P. aeruginosa* were isolated from 350 specimens (29.7%). *Pseudomonas aeruginosa* strains show brilliant green colonies on Cetramide agar that appeared as Gram negative, non- sporing bacilli microscopically, while biochemically positive strains were oxidase positive, Citrate positive, and TSI alkaline (red /red)**.** The positive strains sources were as follows: surgical wound specimens (66 isolates), urine specimens (25 isolates) and burn wound specimens (13 isolates) from different departments.

### Antimicrobial susceptibility pattern of *P. aeruginosa* isolates

There was a high resistance rate among *P. aeruginosa* isolates to the following antimicrobials: Ceftazidime (62.5%), Piperacillin (51%), Meropenem (47.1%), Piperacillin-Tazobactam (46.2%), Amikacin (45.2%) followed by Cefepime (43.3%). However, there were lower resistance rates to Gentamycin (35.6%) and Levofloxacin (34.6%), the lowest resistance was to Colistin (17.3%) as shown in Table 2. Out of 104 *P. aeruginosa* isolates, there were 58 (55.76%) MDR strains resistant to three or more antibiotics of different classes. Supplementary Table S1 contains the results of all MDR isolates against all the tested antibiotics.

**Table 2:** Antimicrobial susceptibility patterns of *P. aeruginosa* isolates

Family	Member	Sensitive	Intermediate	Resistant
Cephalosporin	Ceftazidime	19 (18.3%)	20 (19.2%)	65 (62.5%)
	Cefepime	42 (40.4%)	17 (16.3%)	45 (43.3%)
Aminoglycosides	Gentamycin	58 (55.8%)	9 (8.7%)	37 (35.6%)
	Amikacin	48 (46.2%)	9 (8.7%)	47 (45.2%)
Penicillin	Piperacillin	24 (23.1%)	27 (26%)	53 (51%)
Fluoroquinolones	Levofloxacin	51 (49%)	17 (16.3%)	36 (34.6%)
Carbapenems	Meropenem	37 (33.6%)	18 (17.3%)	49 (47.1%)
Penicillin with beta-lactamase inhibitor	Piperacillin/Tazobactam	35 (33.7%)	21 (20.2%)	48 (46.2%)
Polymyxins	Colistin	69 (66.3%)	16 (15.4%)	18 (17.3%)

### Antibacterial effect of zinc sulfate against *P. aeruginosa* clinical isolates

The antibacterial effect of zinc sulfate against *P. aeruginosa* clinical isolates was tested by broth microdilution method which reported that the MIC of zinc sulfate for all isolates including *P. aeruginosa* PAO1 was 512 µg/ml. Also, the antibacterial effect of serial dilutions of zinc sulfate (512–1 ug/ml concentration) in combination with serial dilutions of the following antibiotics: Ceftazidime, Gentamycin, Levofloxacin, Meropenem, Piperacillin/Tazobactam and Colistin was tested against all resistant isolates to the corresponding antibiotic. Fractional inhibitory concentration FICI is calculated and interpreted. The mean FICI of all tested isolates showed a synergistic effect as shown in Table 3.

**Table 3:** Mean fractional inhibitory concentration (FIC) of antibiotics and Zinc sulfate

Compound	Mean FICA	Mean FICB	Mean FICI	Result
Ceftazidime + Zinc sulfate	0.41	0.0019	0.42	Synergy
Gentamycin + Zinc sulfate	0.34	0.0098	0.35	Synergy
Levofloxacin + Zinc sulfate	0.42	0.008	0.43	Synergy
Meropenem + Zinc sulfate	0.43	0.02	0.45	Synergy
Piperacillin/Tazobactam + Zinc sulfate	0.39	0.01	0.40	Synergy
Colistin + Zinc sulfate	0.28	0.0017	0.28	Synergy

**Fig. 1:**
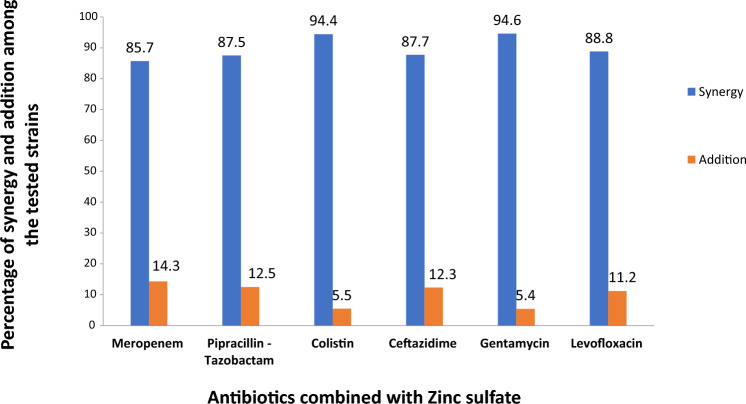
Percentage of isolates with synergistic and additive results to the 6 tested antibiotics in combination with Zinc sulfate

Most of the isolates reported a synergistic result as follows: 94.6%, 94.5%, 88.8%, 87.7%, 87.5%, and 85.7% of the isolates reported a synergistic effect to the following antibiotics: Gentamycin, Colistin, Levofloxacin, Ceftazidime, Meropenem, Piperacillin/Tazobactam with zinc sulfate, respectively. Few isolates reported an additive result for the tested antibiotics with zinc sulfate, respectively (Fig. 1). No antagonistic effect was recorded at all.

### Phenotypic detection of efflux pumps by cartwheel assay in presence and absence of zinc sulfate

**Fig. 2:**
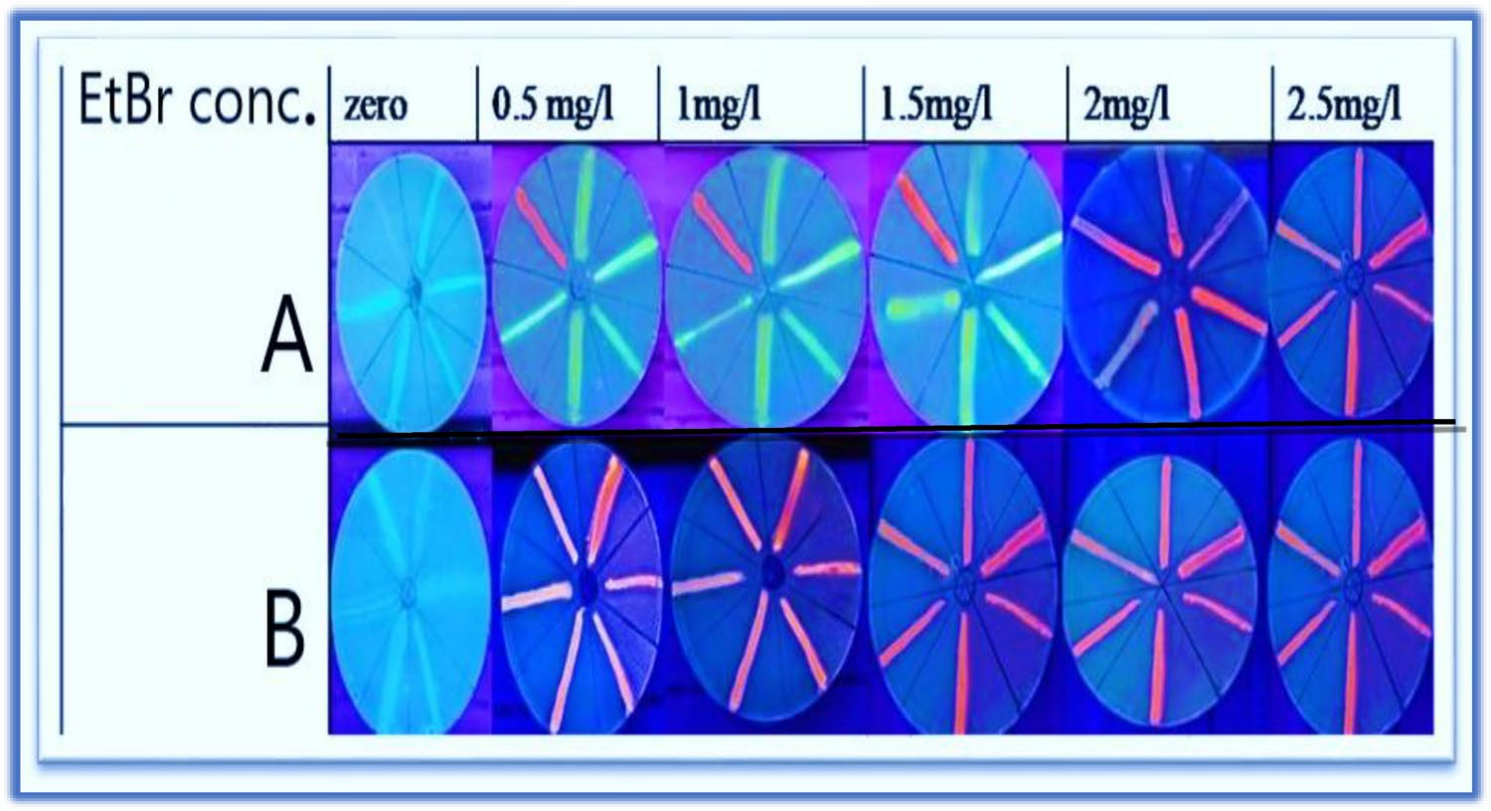
Efflux pump detection by cartwheel assay. **A** and **B** cartwheel assay in the absence and presence of Zinc sulfate respectively, using different concentrations of EtBr. *P. aeruginosa* isolates showed no fluorescence (positive efflux) and appeared grayish-white or yellowish, and *P. aeruginosa* isolates showed fluorescence (negative efflux) that appeared red or orange. In the presence of Zinc sulfate, all *P. aeruginosa* isolates showed fluorescence at all EtBr concentrations

The cartwheel test was used to detect and evaluate the efflux pump activity of MDR *P. aeruginosa* (N = 58) isolates and *P. aeruginosa* PAO1 in the presence and absence of zinc sulfate. This test used two sets of TSA agar plates containing EtBr concentrations of 0, 0.5, 1, 1.5, 2, and 2.5 mg/l. zinc sulfate (sub-MIC, 128 µg/ml) was added to one set of plates. The results of the cartwheel assay conducted before the application of zinc sulfate revealed that 48(82.8%) out of 58 MDR *P. aeruginosa* clinical isolates were positive for the efflux pump. Based on the lowest EtBr concentration at which colonies showed no fluorescence**,** the efflux pump activity was further classified as mild, moderate, or highly active as shown in Table 4. Zinc sulfate inhibited efflux pump activity in all examined MDR *P. aeruginosa* isolates (100%) including *P. aeruginosa* PAO1 strain at all tested EtBr concentrations. This was evidenced by the ability of the bacteria to produce fluorescence at all EtBr concentrations. In the presence of zinc sulfate, all isolates were unable to expel EtBr, even at low concentrations, as shown in Fig. 2.

**Table 4:** Evaluation of efflux pump activity at varying Ethidium bromide concentrations by cartwheel test

EtBr conc. (mg/l)	No. of isolates with positive efflux	Efflux activity
0.5–1	2	Mild (+)
1.5	16	Moderate (+ +)
2	28	Highly expressed (+ + +)
2.5	2	Highly expressed (+ + + +)

### Genotypic detection of efflux pump genes and their regulators by conventional PCR

MDR *P. aeruginosa* isolates were assessed for the presence of genes encoding efflux pumps and their regulators using conventional PCR (Supplementary Fig. S1). The frequencies of the tested efflux pump genes and their regulators among the MDR *P. aeruginosa* isolates in this study are summarized in Table 5. All MDR *P. aeruginosa* isolates tested by conventional PCR were positive for five or more efflux pump genes. The phenotypic and genotypic characteristics of all MDR *P. aeruginosa* isolates are provided in Supplementary Table S1.

**Table 5:** Number and percentage of different efflux pump genes positive isolates

Efflux pump	Tested gene	No. (%) of positive isolates
*MexAB-OprM*	*mexA*	37 (63.8)
	*mexB*	44 (75.9)
	*oprM*	20 (34.5)
Regulator (-ve)	*mexR*	49 (84.50)
*MexCD-OprJ*	*mexC*	34 (58.6)
Regulator(-ve)	*nfxB*	49 (84.5)
*MexEF-OprN*	*mexE*	47 (81)
Regulator(+ve)	*mexT*	45 (77.6)
*MexXY- OprM*	*mexX*	31 (53.4)
	*mexY*	42 (72.4)
Regulator(-ve)	*mexZ*	43 (74.1)
Carbapenem porin	*oprD*	38 (65.55)

### Expression of efflux pumps encoding genes and their regulators in *P. aeruginosa* isolates in the presence and absence of zinc sulfate

Relative expression of efflux pumps encoding genes and their regulators in the presence and absence of zinc sulfate on all MDR *P. aeruginosa* isolates (*n* = 58) were evaluated by real-time RT-PCR.

**Fig. 3:**
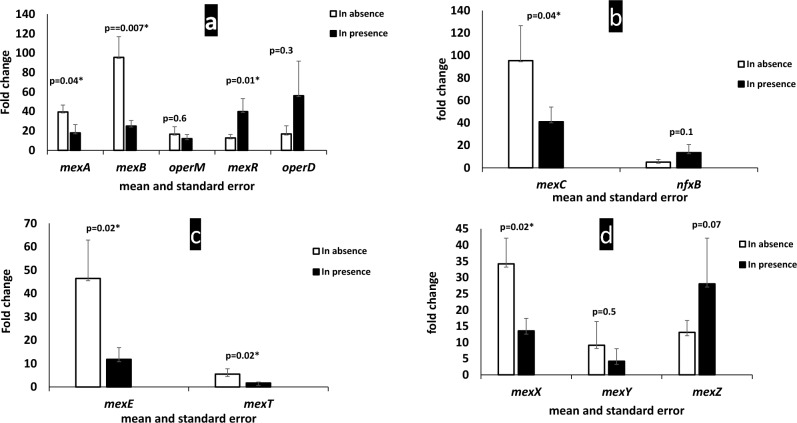
Mean fold change of the efflux pumps encoding gene expression in absence and in presence of Zinc sulfate. **a** Expression of MexAB-OprM *and* OprD pump genes with the negative regulator gene, *mexR* in absence and in presence of Zinc sulfate*.* **b** Expression of *mexC gene* encoding MexCD-OprJ pump with the negative regulator *nfxB* in absence and in presence of Zinc sulfate*.* **c** Expression of *MexE* gene encoding MexEF-OprN pump with the positive regulator, *mexT* in absence and in presence of Zinc sulfate. **d** Expression of *MexX, and mexY genes* encod*e MexXY- OprM* pump with the negative regulator *mexZ* in absence and in presence of Zinc sulfate*.* A significant level of P-value is < 0.05, P-value of means was calculated by using the Wilcoxon test

The results demonstrated the down expression of the efflux pump encoding genes (*mexA, mexB,* and *oprM*) and an increase in the expression of the regulator genes, *mexR* and *oprD* genes after treating the isolates with zinc sulfate in all the 58 MDR isolates as presented in Fig. 3a. The mean fold change in the expression of mexA, mexB, and mexR genes before and after treatment with zinc sulfate in all isolates showed statistically significant differences (P-value < 0.05). In contrast, the differences in the expression of oprM and oprD genes were not significant (P-value > 0.05).

The MexC gene, which encodes the MexCD-OprJ pump, exhibits a significant downregulation (P-value < 0.05). In contrast, the nfxB gene, a negative regulator of this pump, shows a non-significant upregulation (P-value ˃ 0.05) following the application of zinc sulfate, as demonstrated in Fig. 3b.

The mexE gene, which encodes the MexEF-OprJ pump, and the MexT gene, a positive regulator of this pump, both exhibited downregulations following treatment of the isolates with zinc sulfate. However, the difference in mean fold change was statistically significant for the mexE gene (P-value < 0.05), but not for the mexT gene (P-value > 0.05), as shown in Fig. 3c. The expression of the MexXY-OprN pump was assessed by measuring the expression of the mexX and mexY genes, as well as the negative regulator gene, mexZ. Both mexX and mexY genes showed downregulation after zinc sulfate treatment, while mexZ gene expression was upregulated. The difference was significant for the mexX gene (P-value < 0.05), but not for mexY and mexZ (P-value > 0.05), as shown in Fig. 3d.

## Discussion

This study analyzed 104 *P. aeruginosa* clinical isolates from 350 different specimens, including surgical wound, urine, and burn wound samples, to assess their antimicrobial susceptibility. The highest resistance rates were observed for Ceftazidime and Piperacillin, followed by Meropenem, Piperacillin-Tazobactam, Amikacin, Cefepime, Gentamycin, and Levofloxacin. Colistin exhibited the lowest resistance rate.

Although carbapenems were considered the most effective treatment for MDR *P. aeruginosa* infections, resistance to these drugs has risen due to the activation of β-lactamases and efflux pumps. The high resistance rate to Meropenem may also be associated with the selective pressure from the increased use of carbapenems in treating *P. aeruginosa* infections. Our findings revealed that colistin was the most effective antibiotic against *P. aeruginosa,* with a 17.3% resistance rate This result was aligns with Abavisani as he reported that colistin resistance rate 17.1% (Abavisani et al. 2021). However, there is some concern regarding the use of colistin to treat *P. aeruginosa* infections due to its potential neurological and nephrotoxic effects (Bonyadi et al. 2022)***.*** This study found that 55.76% of isolates were MDR which means they are resistant to three or more antibiotics of different classes, this percentage was lower than that of Abbas et al. and Edward et al. in Egypt who recorded that 79.8% and 100% of the studied *P. aeruginosa* isolates were MDR, respectively (Abbas et al. 2018; Edward et al. 2023). The high MDR rates may result from antibiotic misuse and the emergence of resistant strains, highlighting the need for strict prescription guidelines to address the problem.

This study demonstrated that zinc sulfate, both alone and in combination with antimicrobials, exhibits a strong antibacterial effect. Our findings indicated that zinc sulfate MIC against *P. aeruginosa* isolates was 0.5 mg/ml. A similar study by Abdelkader and Al-Saedi tested the antimicrobial effect of different concentrations of zinc sulfate against *P. aeruginosa* isolates (2–20 mg/ml) by the agar well diffusion technique reported a good antimicrobial effect of all zinc sulfate concentrations with a zone of inhibition between 13.5 and 20.5 mm (Abdalkader and Al-Saedi 2020). In this study, zinc sulfate exhibited a synergistic effect when combined with various antibiotics against most of the *P. aeruginosa* isolates, with only a few isolates showing an additive effect. The strongest synergistic activity was observed with gentamicin and colistin, as 94.6% and 94.5% of the isolates showed a synergistic response, respectively. Our findings not only confirmed the antimicrobial activity of zinc sulfate but also highlighted its synergistic potential when paired with different antibiotics. This suggests that zinc sulfate could serve as an effective antibiotic adjuvant in treating *P. aeruginosa* infections, potentially helping to reduce antibiotic resistance in this bacterium.

Efflux pumps are key mechanisms of both intrinsic and acquired resistance in bacteria, as they enable the removal of toxic substances, including antibiotics, medications, pollutants, and the cell’s own secreted products (Al Rashed et al. 2020). Efflux pumps in *P. aeruginosa* are frequently associated with their MDR patterns. In this study, all MDR *P. aeruginosa* isolates were examined phenotypically and genotypically for the presence of efflux pumps. The phenotypic activity of efflux pumps was observed in 82.7% of MDR *P. aeruginosa* clinical isolates by cartwheel assay at 0.5 mg/l EtBr concentration, this result was higher than that of Bhivina et al. who reported that 60% of the tested isolates showed efflux pump activity by cartwheel assay at 0.5 mg/l EtBr concentration (Bhivina 2017). The efflux pump activity was further categorized into mild, moderate, or highly active based on the minimal concentration of EtBr at which the isolates showed no fluorescence, Osman et al. also reported that EtBr can be effluxed in *P. aeruginosa* isolates at different concentrations, depending on the degree of efflux pump activity (Osman et al. 2018)***.*** Additionally**,** we assessed all MDR *P. aeruginosa* clinical isolates for the presence of efflux pump genes by conventional PCR. MexAB- OprM efflux pump genes were reported to be harbored by 63.8%, 75.9%, and 34.5% of MDR *P. aeruginosa* isolates for *mexA*, *mexB,* and o*prM,* respectively. This is following Abd AL-Zwaid et al. who found that 83.54%, 63.29%, and 48.1% of MDR *P. aeruginosa* isolates were positive for *mexA, mexB,* and *oprM,* respectively (Abd AL-Zwaid and Al-Dahmoshi 2022).

The regulator gene of the MexAB-OprM efflux pump, mexR, was present in 84.5% of MDR *P. aeruginosa* isolates, which is closely aligned with the findings of Abd Al-Zwaid et al., who reported that 89% of their isolates contained the mexR gene (Abd al-zwaid and Al-dahmoshi 2022)***.*** Resistance to carbapenem is often produced in *P. aeruginosa* strains by the deletion of the outer membrane protein *OprD* or the overexpression of efflux pumps, particularly MexAB-OprM (Hazirolan and Özkul 2023). In this study, the *oprD* gene was detected in 65.5% of MDR *P. aeruginosa* isolates, a higher percentage than the 33.3% reported by Saleh and Motib, this may explain the high resistance to meropenem observed in our study (Saleh and Motib 2023).

In this study, MexCD-OprM was assessed through the *mexC* gene and *nfxB,* the inhibitory regulator of this pump. We reported that 58.6% and 84.5% of MDR *P. aeruginosa* isolates had *mexC* gene and *nfxB,* respectively and These results are lower than the result recorded by Quddus et al. who recorded that both genes presented in 89% of their isolates (Quddus et al. 2023)***.***

In this study, we examined one of the MexEF-OprN pump genes, mexE, which was positive in 81% of MDR *P. aeruginosa* isolates. The regulator gene for the MexEF-OprN pump, mexT, was positive in 77.6% of the MDR isolates. For the identification of the MexXY-OprM pump, we found that 53.4% and 72.4% of MDR *P. aeruginosa* isolates harbored mexX and mexY, respectively. These findings are lower than those reported by Al Saadi, who found that 100% of isolates contained mexY (Alsaadi 2022).

In this study, all MDR *P. aeruginosa* isolates examined by conventional PCR were found to be positive for one or more efflux pumps. The study also reported that the specificity and sensitivity of the cartwheel assay, compared to conventional PCR for detecting efflux pumps, were 100% and 82.7%, respectively. These findings are consistent with those of Bhivina, who reported 100% specificity and 88.2% sensitivity for the cartwheel assay (Bhivina 2017)***.***

In this study, we have evaluated zinc sulfate as an efflux pump inhibitor, in this regard MDR *P. aeruginosa* isolates have been evaluated for their efflux pump activity by Cartwheel assay in the presence of 1/4 MIC of zinc sulfate (125 µg/ml). In all tested MDR *P. aeruginosa* isolates, zinc sulfate could suppress efflux pump activity, it demonstrated by the ability of the isolates to produce fluorescence at all doses of Ethidium bromide in the presence of 1/4 MIC of zinc sulfate. This work is a novel study to examine the zinc sulfate inhibitory effect on efflux pumps.

Also, we have tested the effect of zinc sulfate on efflux pump gene expression by real-time PCR. The results demonstrated down expression of the efflux pump genes (*mexA, mexB, oprM*, *mexC, mexE, mexX,* and *mexY*) after treating the isolates with zinc sulfate, however, significant down-regulation was recorded with *mexA, mexB, mexC, mexE,* and *mexX genes* (P-value < 0.05) only. The current study also investigated the expression levels of Efflux pump regulator genes before and after the application of zinc sulfate. The expression of the MexAB-OprM operon is strictly controlled by many regulators, including *mexR*, a repressor gene, this study reported that the* mexR* gene showed significant upregulation in its expression after treating the isolates with zinc sulfate (P-value = 0.01). It was previously reported that down expression of the outer membrane protein *OprD,* a carbapenem-specific porin, resulted in lower susceptibility of *P. aeruginosa* to Meropenem (Hazirolan and Özkul 2023), in our study, it was upregulated by zinc sulfate increasing its expression, and increasing the susceptibility of *P. aeruginosa* isolates to Meropenem (P-value = 0.3). Our study reported that there was a non-significant down expression of *the mexT* gene. a transcriptional activator of the MexEF-OprN efflux pump after the addition of zinc sulfate (P-value = 0.1). The *nfxB and mexZ* genes, repressors of MexCD-OprJ and MexXY-OprM, respectively, were non-significantly upregulated after the addition of zinc sulfate to the tested isolates (P-value = 0.1 and 0.2, respectively).

This research is considered the first to report the efflux pump inhibitory effect of zinc sulfate and the corresponding synergistic effect with antibiotics against *P. aeruginosa* tested isolates. which supports that zinc sulfate should be used as an adjuvant with antibiotics to suppress the efflux pump activity, which is a crucial mechanism of antibiotic resistance.

## Conclusions

In conclusion, multidrug-resistant (MDR) *P. aeruginosa* infections are widespread in Minia University Hospitals. The high incidence of MDR strains is concerning, highlighting the urgent need for the development of new antimicrobial agents to combat resistance. The combination of zinc sulfate and antibiotics produces a synergistic effect, demonstrating its significant antimicrobial action against *P. aeruginosa*.

We recommend conducting further in vivo studies to assess the potential side effects of zinc sulfate. Additionally, extensive molecular research involving multiple bacterial isolates should be carried out to examine the impact of zinc sulfate on virulence factors and the expression of antibiotic resistance genes.

## Supplementary Information

Below is the link to the electronic supplementary material.

Supplementary file1 (DOCX 226 KB)

## Data Availability

All data generated or analyzed during this study are included in this article.
