# Supplementary material for: Zinc sulfate acts as an efflux pump inhibitor on Pseudomonas aeruginosa clinical isolates
Source: World J Microbiol Biotechnol. 2025 Apr 28;41(5):139. doi: 10.1007/s11274-025-04352-4 (PMC12034582; doi:10.1007/s11274-025-04352-4)
Supplement: Supplementary file 1 — Supplementary file1 (DOCX 226 KB) [file 11274_2025_4352_MOESM1_ESM.docx]

**Supplementary Table S1: Phenotypic and genotypic characterization of MDR isolates**

| No. | Specimen | Dep. | MDR pattern | C. test | *MexAB-OprM* | *mexR* | *mexC* | *nfxB* | *MexE* | *mexZ* | *MexXY* | *mexT* | *oprD* |
| --- | --- | --- | --- | --- | --- | --- | --- | --- | --- | --- | --- | --- | --- |
| 10 | Urine | Gyna | CAZ, CPM, CN, AK, PRL, MER, TAZ | + | ++ | + | + | + | + | + | + | + | + |
| 11 | Urine | Gyna | CAZ, CPM, CN, AK, PRL | + | +++ | + | + | + | + | + | ++ | + | - |
| 65 | Urine | Gyna | CAZ, CN, AK, PRL, MER | + | + | - | + | - | + | + | + | + | + |
| 66 | Urine | Gyna | CAZ, CPM, CN, AK, PRL | + | + | + | + | + | + | + | + | + | + |
| 67 | Urine | Gyna | CAZ, CPM, CN, AK  PRL, MER, COL | + | + | + | + | - | + | + | + | + | + |
| 72 | Urine | Gyna | CAZ, CN, AK, PRL, LEV | + | ++ | + | - | + | + | + | ++ | + | - |
| 73 | Urine | Gyna | CAZ, CPM, CN  AK, PRL, LEV | + | + | - | - | + | + | + | + | + | - |
| 78 | Wound | Plastic | CAZ, CPM, CN, AK, PRL, MER, LEV | + | ++ | + | + | - | - | + | - | + | + |
| 82 | Burn | Plastic | CAZ, CPM, CN  AK, PRL, LEV | + | + | + | + | - | + | + | + | + | - |
| 84 | Burn | Plastic | CAZ, CPM, AK, PRL, MER, TAZ | - | + | + | - | + | - | + | + | + | + |
| 88 | Burn | Plastic | CAZ, CPM, AK, PRL, MER, TAZ | + | + | - | - | + | + | + | + | + | + |
| 89 | Wound | Plastic | CAZ, CPM, CN, AK, PRL, TAZ, COL | + | ++ | + | - | + | + | + | ++ | + | - |
| 95 | Wound | Plastic | CAZ, CN, AK, PRL, TAZ, COL | + | + | + | + | + | + | + | + | + | + |
| 96 | Wound | Plastic | CAZ, CPM, CN, AK, PRL | - | ++ | + | - | + | - | + | - | + | + |
| 97 | Wound | Plastic | CAZ, CN, AK, PRL,  MER | + | ++ | + | + | + | + | + | + | + | + |
| 100 | Wound | Plastic | CAZ, CN, AK, PRL,  MER | + | +++ | + | - | + | - | + | ++ | + | + |
| 102 | Wound | Plastic | CAZ, CPM, AK, PRL, LEV, TAZ, COL | + | ++ | - | + | + | + | - | ++ | - | + |
| 108 | Burn | Plastic | CAZ, CN, AK, PRL, MER | + | ++ | + | + | + | + | - | ++ | + | + |
| 111 | Wound | Plastic | CAZ, CPM, PRL, MER, TAZ, COL | + | ++ | + | - | + | + | - | ++ | + | + |
| 119 | Wound | General | CAZ, CN, AK, PRL, MER | + | ++ | + | - | + | + | + | ++ | + | + |
| 131 | Wound | General | CAZ, CPM, CN, AK,  PRL, LEV | + | ++ | + | + | + | + | + | ++ | + | - |
| 140 | Burn | Plastic | CAZ, CPM, CN, AK, PRL, LEV, MER, TAZ | - | +++ | + | + | + | + | + | ++ | + | + |
| 142 | Wound | Plastic | PRL, LEV, MER, COL | - | ++ | + | - | + | + | + | ++ | + | + |
| 143 | Wound | Plastic | CAZ, CPM, CN, AK,  PRL, LEV, MER | + | + | - | + | + | + | + | + | + | + |
| 147 | Wound | Plastic | CAZ, CPM, CN, PRL, MER,  TAZ | + | + | + | + | + | + | - | + | + | + |
| 148 | Wound | Plastic | CAZ, CN, AK, PRL, TAZ, COL | + | + | - | + | + | + | - | ++ | - | + |
| 162 | Burn | ICU | CPM, MER, COL | + | +++ | + | + | + | + | + | ++ | + | + |
| 167 | Wound | Plastic | CAZ, CPM, PRL, LEV, MER | - | ++ | + | + | + | + | + | ++ | - | + |
| 168 | Wound | Plastic | CAZ, CPM, AK,  LEV, MER | + | + | + | + | + | + | + | + | + | + |
| 170 | Wound | Plastic | CAZ, CPM, LEV, MER, TAZ | + | ++ | + | + | + | + | - | + | - | + |
| 171 | Burn | ICU | CAZ, CPM, CN, AK, PRL, LEV | + | + | + | - | + | + | + | ++ | - | - |
| 177 | Wound | ICU | MER, TAZ, COL | - | ++ | - | - | - | + | - | + | + | - |
| 182 | Wound | General | CAZ, CPM, CN, AK, PRL, LEV, MER | + | + | + | + | - | - | + | + | - | + |
| 212 | Wound | ICU | PRL, MER, TAZ | + | + + | + | + | + | - | + | + | + | - |
| 213 | Wound | ICU | CAZ, CPM, CN, AK,  PRL, LEV, MER, COL | + | + | + | + | + | + | + | ++ | - | + |
| 185 | Wound | ICU | AK, MER, TAZ, COL | + | + | + | + | + | + | - | + | + | - |
| 190 | Wound | Plastic | CAZ, CPM, AK, MER | + | + | + | - | + | + | + | + | + | - |
| 196 | Wound | Plastic | CAZ, CPM, PRL, LEV, MER, TAZ | + | ++ | + | - | + | + | + | ++ | + | - |
| 198 | Wound | Plastic | CAZ, PRL, MER, TAZ | + | + | + | - | + | - | + | ++ | + | - |
| 214 | Wound | Plastic | CAZ, MER, TAZ, COL | - | ++ | + | - | + | + | + | - | + | + |
| 215 | Wound | Plastic | CAZ, CPM, CN, AK,  PRL, LEV | + | - | + | - | + | + | + | + | + | - |
| 226 | Urine | ICU | CAZ, CPM, PRL, LEV, TAZ | + | + | + | + | + | - | - | + | - | - |
| 229 | Wound | Plastic | CAZ, CPM, AK, PRL, LEV, TAZ, COL | + | +++ | + | + | - | + | - | - | + | + |
| 230 | Wound | Plastic | CAZ, CPM, AK, PRL, LEV, MER, TAZ, COL | + | ++ | + | + | + | + | + | + | + | + |
| 231 | Wound | Plastic | CAZ, CN, AK, PRL, LEV, MER, TAZ | + | +++ | + | + | + | + | + | + | - | + |
| 233 | Urine | ICU | CAZ, CPM, CN, AK,  PRL, LEV, TAZ | - | + | + | - | + | - | - | + | + | + |
| 234 | Urine | ICU | CAZ, CPM, AK, PRL, LEV | - | ++ | + | - | + | + | - | + | + | - |
| 241 | Wound | Plastic | CAZ, CPM, PRL, LEV, MER, TAZ | + | ++ | + | - | + | + | - | ++ | - | + |
| 242 | Wound | Plastic | CAZ, PRL, LEV, MER, TAZ | + | +++ | + | + | + | + | + | ++ | - | + |
| 245 | Wound | ICU | CAZ, AK, MER, TAZ, COL | + | ++ | + | - | - | + | + | - | + | + |
| 246 | Wound | ICU | CAZ, CPM, CN, AK, PRL, LEV, TAZ | + | + + | + | + | + | - | + | + | + | - |
| 258 | Wound | Plastic | CAZ, CPM, CN, AK  PRL, LEV, MER, TAZ | + | + | + | - | + | + | + | + | + | + |
| 260 | Burn | Plastic | LEV, MER, TAZ, COL | + | +++ | + | + | + | + | - | ++ | + | + |
| 261 | Wound | Plastic | LEV, MER, TAZ | - | +++ | + | - | + | + | - | ++ | + | - |
| 263 | Wound | ICU | CPM, CN, AK, PRL, LEV, TAZ, COL | + | ++ | + | - | + | + | + | - | - | - |
| 268 | Wound | Plastic | CAZ, PRL, TAZ | + | + + | - | + | - | + | + | + | + | - |
| 283 | Wound | ICU | CPM, AK, MER | + | - | - | + | + | + | + | - | + | + |
| 284 | Wound | ICU | CAZ, CPM, CN, AK, PRL, MER, TAZ | + | + | + | + | - | - | + | + | - | + |

C test, cartwheel test; Dep, department; Gyna, gynecology; plastic, plastic surgery, and General, general surgery. CAZ(Ceftazidime), CPM(Cefepime), CN(Gentamicin), AK(Amikacin), PRL (Piperacillin), MER (Meropenem), TAZ (Piperacillin/Tazobactam), LEV (Levofloxacin) and COL (Colistin sulfate).

In C test: + (positive), - (negative).

In gene expression: + (mild), ++ (moderate), +++ (highly expressed).


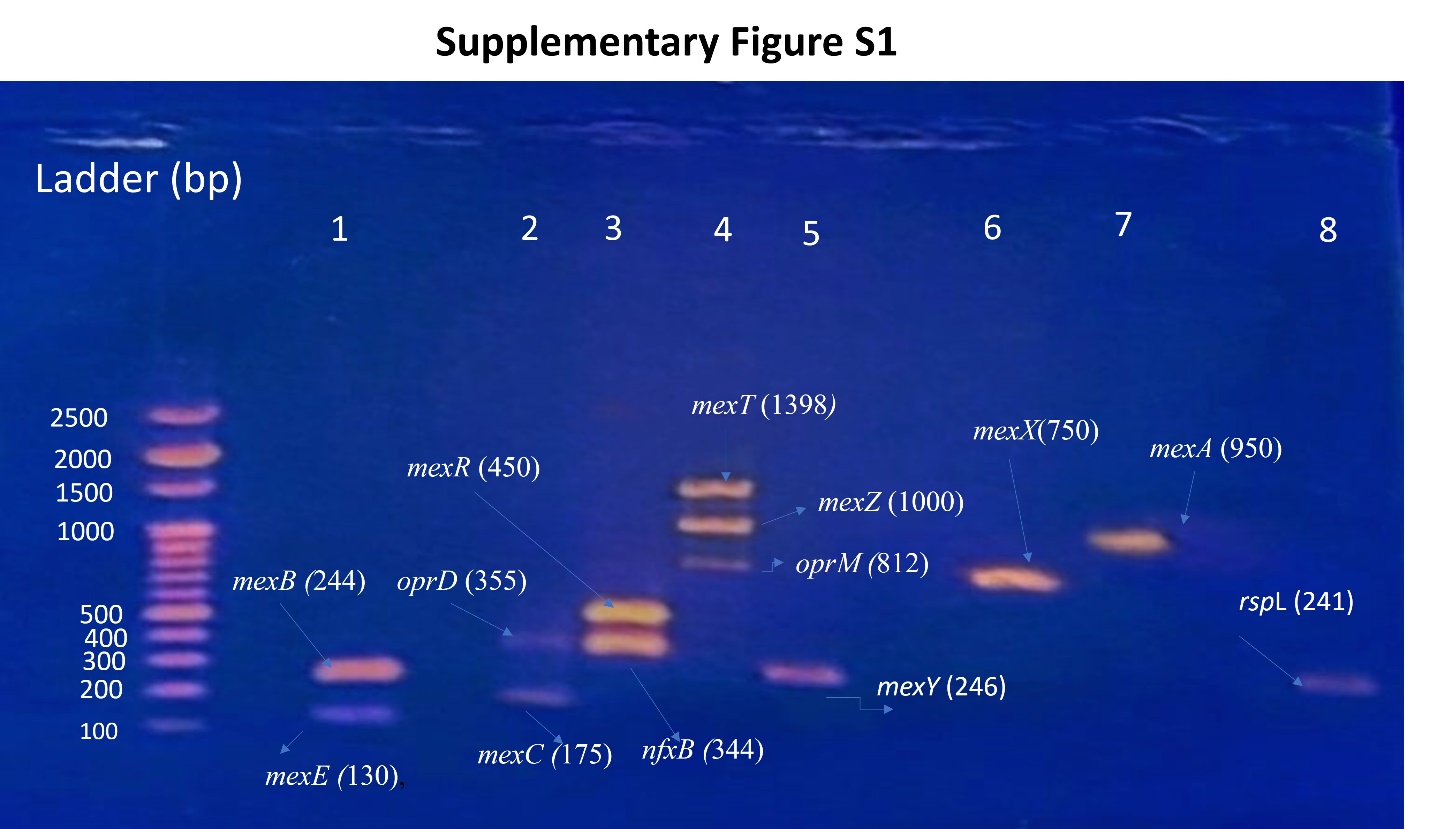


**Supplementary Figure S1: Gel electrophoresis of the PCR amplified products for the tested genes**.

A ladder is a molecular size marker (100-2500bp). Lane 1**:** PCR multiplex of *mexB (*244 bp) and *mexE (*130bp), Lane 2: PCR multiplex of *mexC (*175bp) and *oprD* (355bp), Lane 3: PCR multiplex of *mexR (*450 bp), *nfxB (*344 bp), Lane 4: PCR multiplex of *mexT* (1398bp*), mexZ* (1000bp) and *oprM* (812bp), Lane 5:PCR product of *mexY* (246bp), Lane 6: PCR product of *mexX* (750bp), Lane 7: *mexA* (950bp) and Lane 8: *rspl* (241bp).
